# Suicidal behavior and associated factors among prisoners in Dilla town, Dilla, Ethiopia 2020: An institutional based cross-sectional study

**DOI:** 10.1371/journal.pone.0267721

**Published:** 2022-05-11

**Authors:** Endashaw Habtamu, Defaru Desalegn

**Affiliations:** 1 Department of Psychiatry, College of Health Sciences, Dilla University, Dilla, Ethiopia; 2 Department of Psychiatry, College of Health Sciences, Mettu University, Mettu, Ethiopia; Gachon University Gil Medical Center, REPUBLIC OF KOREA

## Abstract

**Background:**

Suicide is a serious cause of mortality worldwide and is a psychiatric emergency. Among prisoners, it is the leading cause of death compared to the general population. However, suicide in prison is a neglected public health issue especially in middle- and low-income countries including Ethiopia. Therefore, this study aimed to assess the prevalence and associated factors of suicidal behavior among prisoners in Dilla Town Correctional Center, South Ethiopia.

**Methods:**

An institution-based cross- sectional study was conducted from May13- June 13, 2020 in the Dilla Town Correctional Center. The simple random sampling technique was used to select 650 prisoners. Data were collected by face-to—to-face interview. Suicidal ideation and attempts were assessed by using the suicidality module of World Mental Health survey initiative version of the World Health Organization composite International diagnostic interview. Data were coded, entered with EP-data version 3.1, and analyzed by using Statistical Package for Social Science version 24. Multivariate binary logistic regression analysis was used to determine the significant association between explanatory variables and outcome variables at 95% CI.

**The results:**

The prevalence of suicidal ideation and attempt among prisoners were 21.9% (95%CI, 18.4–25.2) and 13.1% (95%CI, 10.6–15.8), respectively. Female sex [(AOR) = 2.6, 95%CI, (1.39, 8.2)], divorced/widowed [AOR = 3.67, 95%CI, (2.05, 6.58)], family history of mental illness [AOR = 2.49, 95%CI, (1.41, 4.38)], common mental disorder [AOR = 1.98, 95%CI, (1.25, 3.16)] and poor social support [AOR = 2.68, 95%CI, (1.42, 5.06)] were statistically associated with suicidal ideation. Whereas, female sex [AOR = 3.24, 95%CI, (1.89, 9.4)], previous incarceration [AOR = 2.38, 95%CI, (1.2, 5.16)], and family history of mental illness [AOR = 2.08, 95%CI, (1.11, 3.9)] were associated with suicide attempt.

**Conclusion:**

The prevalence of suicidal ideation and attempts among prisoners were high. The special attention in early screening and treatment of suicide among prisoners and collaborating with health institutions is important for better management and prevention.

## Introduction

Suicide is an act of intentionally terminating one’s own life and it is a complex process that involves a series of pathways and mechanisms [1]. Suicidal ideation is any self-reported thought of engaging in suicide-related behavior or active thoughts about killing oneself [2]. Suicidal ideation is also an important phase in the suicidal process preceding attempted suicide; which is the major risk factors for completing suicide [3]. A suicide attempt is defined as a self-injurious behavior with a nonfatal outcome accompanied by explicit or implicit evidence that a person intends to die [4].

Suicide is an important public health issue and is the 10^th^ leading cause of death worldwide [5]. In the general population, the annual global suicide rate is reported as 11.4 per 100 000 population and there is one death every 40 seconds [6]. In Ethiopia, the prevalence of suicidal ideation and attempted suicide among general population were ranged from 1 to 55% and 0.6% to 14% respectively [7].

Prisoners are special populations that are found under court control with limited liberty, autonomy, and communication with family and friends [8]. An estimated 10.1 million people are in correctional institutions worldwide and the majority of them living in low and middle-income countries (LAMIC) [9]. According to World Prison Population List report 2014, in Ethiopia, about 113,727 peoples are found in prison [10]. Prisoners have poor general health and high rates of physical illness as well as relatively high rates of mental disorder [9]. Prisoners are a vulnerable group with a nine-fold increase of suicidal risk and a twofold increase of suicide rate when compared to the general population [11].

As different studies indicated, the characteristics of the prison setting itself may increase the risk of suicide [12]. Suicidal behavior is the most common cause of death in correctional institutions and it is an international problem with prisoners [13].

Globally, the rate of suicide is found in the range of 16–19 deaths per 100,000 each year among prisoners [14]. A meta-analysis conducted in the United Kingdom among prisoners found that the risk of suicide was 15 times higher [13]. Suicide is also the third leading cause of death in US correctional institutions and the second in jails, after natural causes and Acquired immune deficiency syndrome (AIDS) [15]. Suicide in prison occurs mainly at the beginning of incarceration, 25% during the first 2 months, and 50% in the first 6 months [16]. The is related to the difficulty in adapting the prison environment, the deprivation of liberty, and humiliation due to the disclosure of a committed crime in front of family and society [17]. Even though suicide is common in developing and developed countries in a correctional setting, little attention is paid to it outside of mental health or psychiatric settings, particularly in our country.

The presence of suicidal ideation and attempts among prisoners has consequence in the social, emotional, and economic aspects of family, friends, and community and might lead to an unsuspected end of an individual’s life [18]. Besides, the burdens and results of the problems, suicidal ideation and endeavor among these bunches of individuals have not been well investigated in Ethiopia. As a result, considers on this range might give basic information for the government and correctional institutions to alarm for early discovery and mediations of the issues. Therefore, the aim of this study was to determine whether there was a link between suicidal behavior and socio-demographic characteristics, clinical characteristics, and social support among prisoners in southern Ethiopia.

## Methods and materials

### Study design

An institutional-based cross-sectional study design was conducted from May 13 -June13/ 2020.

### Study setting

The study was conducted in the Dilla Town correctional center, which is found in SNNP region and is 359 kilometers (km) away from Addis Ababa, the capital city of Ethiopia. According to the 2007 National Census Report, Dilla town has a total population of 216,434, of whom 124,742 are men and 91,692 women, [19]. The correctional center was established in 1954 and it gives service to a large number of populations. There are more than 1500 sentenced prisoners for different types of crimes.

### Study subjects

All prisoners at the Dilla town correctional center were considered as source population. The study populations were sample of prisoners at the Dilla town correctional center, who avail during the data collection period and included in the study. Prisoners who were critically ill at the time of data collection and unable to communicate (unable to speak and unable to hear) were excluded from the study.

### Sampling procedure

The minimum the sample size required for this study was determined by using the formula to estimate single population proportion, n = ((z ᾳ/2)2p(1-p))/d2 by using the following assumptions: the prevalence of suicidal ideation among prisoners at Jimma Correctional Center southwest Ethiopia was 16.6% [20], 95% Confidence Interval and 3% margin of error. Finally, after adding 10% nonresponse rate, the final sample size was 640. A simple random sampling technique was used to select the study population. To select the study population by the sampling frame, a computer generated random number identifying the frame lists of prisoners was used in a simple random sampling technique.

### Data collection instruments

Social support was collected by using Oslo-3 items social support scale, which commonly used to assess social support and it has been used in several studies. The score ranges from 3–14 and has 3 categories: poor support 3–8, moderate support 9–11, and strong support 12–14 [21]. The Oslo-3 item scale has internal consistency (Cronbach alpha’s (α = 0.84) in this study.

Perceived stigma was assessed by Jacoby 3 item perceived stigma scale with internal consistency of Crobach alpha’s (α = 0.71) and it has three item questions which were used to assess the individual’s perception of stigma regarding their illness [22]. Each of the three questions had two possible answers and scored 0 for “no” responses and 1 for “yes” responses. To say the patient is stigmatized, the sum score should be 1 and above. The internal consistence (Cronbach alpha) of 3-item perceived stigma scale in the current study is 0.77.

Common Mental disorders were measured by self -reporting questionnaire (SRQ-20), which was developed by the World Health Organization (WHO). The screening instrument includes 20 yes/no questions on depression, anxiety, somatic symptoms experienced in the last 30 days. The Self Reporting Questionnaire (SRQ-20) has been tested in numerous settings. Depending on the setting, community surveys or primary care, a varied cutoff has been used with cutoff point 6 was found to have a specificity of 83.7% and sensitivity 84.8% [23]. Its internal consistence (Cronbach alpha) in the current study is 0.91.

Suicidal ideation and attempts were assessed by using the suicidality module of World Mental Health (WMH) survey initiative version 3.0 of the World Health Organization (WHO) composite International diagnostic interview (CIDI). It was validated in Ethiopia both in clinical and community settings and its internal consistency and percentage of agreement was 0.78 and 93.2%-100% of full scale [24]. The respondent who answers yes, to the question “Have you ever seriously thought about committing suicide?” were considered as having suicidal ideation, and the respondents who answer yes, to the question “Have you ever attempted suicide?” were considered as have suicide attempt. Its internal consistence (Cronbach alpha) in the current study is 0.89.

### Data quality assurance

The questionnaire was designed and modified appropriately and was translated to the local language (gedofa) and (Amharic) national language of Ethiopia to be understood by all participants and translated back to English. Pre- test was done about 5% (33 participants) of the total sample. Data collectors were supervised daily and the filled questionnaires were checked for completeness and consistency by the supervisor and principal investigator daily.

### Data analysis procedure

Data were coded, entered with EPI-data version 3.1, and analyzed by using Statistical Package for Social Science (SPSS version 24). Descriptive statistics such as (frequency, percentage, and mean) were computed and presented using tables, charts; and figures show pictures of the data. Bivariate logistic regression analysis was performed to determine each of the explanatory variables and variables with p- value less than 0.2 during the bivariate analysis were entered into multivariable analysis. Multivariable logistic regression analysis was conducted to determine the presence of a statistically significant association between explanatory variables and outcome variables. P values less than 0.05 were considered statistically significant and the strength of the association was presented by odds ratio with 95% confidence interval (CI).

### Ethical consideration

Ethical clearance was obtained from the ethical review committee of Dilla University. Formal letter of permission was obtained from this office and submitted to Dilla Town Correctional Center. Written informed consent was obtained from study participants before interview. The right to refuse or discontinue participation at any time they want and the chance to ask any thing about the study was given. All personnel information was kept anonymously and confidentiality was assured throughout the study period.

## Results

### Socio-demographic characteristics

A total of 640 participants were interviewed with a response rate of 98.5%. The median age of the study participants was 28 years with interquartile range (IQR) of 11 years. Most of the study subjects were in the age group of 18–27 years old, 287 (44.8%). The majority of the participants, 578 (90.3%) were males, 418 (65.3%) were urban residency, 635 (99.2%) were Gedeo ethnic group, 623 (97.3%) were Orthodox by religion and 326 (50.9%) were single in marital status. Some of the participants, 286 (44.7%) had primary school education, 245 (38.3%) were farmers by occupation, 219 (34.2%) had done theft and robbery of crime followed by 202 (31.6%) murders (**Table 1**).

**Table 1:** Frequency distribution of socio-demographic characteristics of prisoners at Dilla town correctional center, south, Ethiopia, 2020 (n = 640).

Variable	Categories	Frequency	Percentage (%)
**Age**	**18–27**	287	44.8
	**28–37**	223	34.8
	**38–47**	92	14.4
	**>47**	38	6.0
**Sex**	**Male**	578	90.3
	**Female**	62	9.7
**Residence**	**Urban**	418	65.3
	**Rural**	222	34.7
**Religion**	**Orthodox**	623	97.3
	Others*	17	2.7
**Marital Status**	**Single**	326	50.9
	**Married**	216	33.8
	**Divorced/Widowed**	98	15.3
**Ethnicity**	**gedeo**	635	99.2
	**Other** **	5	0.8
**Educational Level**	**non-educated**	137	21.4
	**Educated**	503	78.6
**Occupation**	**Employed**	79	12.3
	**Trading**	65	10.2
	**Farmer**	245	38.3
	**Student**	53	8.3
	**Unemployed**	172	26.9
	**Other** ***	26	4.0
**Type of Crime**	**Theft & robbery**	219	34.2
	**Murderer**	202	31.6
	**Rape**	35	5.5
	**Corruption**	30	4.7
	**Physical attack**	128	20.0
	**Other** ****	26	4.0

*Others = Muslim, Protestant

**Others = Tigray, Oromo

***Others = House wife, Daily laborer, and driver

****Other crimes = Emotional attack, cheating.

### Clinical related characteristics

Regarding the clinical related characteristics, 36 (5.6%) of the respondents had a family history of suicide attempt and 23 (3.6%) had a family history of suicide. Of the respondents, 78 (12.2%) had a family history of mental illness. The study also revealed that 75 (11.7%) of the respondents had a history of mental illness. More than half of the respondents 390 (60.9%) had common mental disorders. Moreover 82 (12.8%) of the respondents had a chronic physical illness **(Table 2)**

**Table 2:** Description of clinical factors among prisoners at Dilla town correctional center, south, Ethiopia, 2020 (n = 640).

Variable	Categories	Frequency	Percentage (%)
**Family history of suicide attempt**	**Yes**	36	5.6
	**No**	604	94.4
**Family history of suicide commit**	**Yes**	23	3.6
	**No**	617	96.4
**Family history of mental illness**	**Yes**	78	12.2
	**No**	562	87.8
**History of mental illness**	**Yes**	75	11.7
	**No**	565	88.3
**Common mental disorder**	**Yes**	390	60.9
	**No**	250	39.1
**Chronic physical illness**	**Yes**	82	12.8
	**No**	558	87.2
**Types of chronic physical illness**	**Hypertension**	31	37.8
	**Diabetic mellitus**	22	26.8
	**Cardiac disease**	15	18.3
	**Others** ****	14	17.1

****Others: HIV/AIDS, Asthma, Tuberculosis.

### Prison and substance related characteristics

Concerning to prison-related characteristics of the participants, 287 (44.8%) of the participants had <1 year length of stay, 51 (8.0%) were in prison for more than six years. 46 (7.2%) of the participants had a history of previous incarceration, 39(6.1%) were incarcerated in the past, More than half of the 354(55.3%) reported that they were ever alcohol users, 66(10.3%) of the respondents were ever cigarette smokers **(Table 3).**

**Table 3:** Description of prison and substance related factors among prisoners at Dilla town correctional center, south, Ethiopia, 2020 (n = 640).

Variable	Categories	Frequency	Percentage (%)
Length of stay	<1 year	287	44.8
	1-3years	164	25.6
	4-6years	138	21.6
	>6 years	51	8.0
Pervious incarceration	Yes	46	7.2
	No	594	92.8
Number of Pervious incarceration	Once	39	84.8
	Twice	7	15.2
Ever alcohol use	Yes	354	55.3
	No	286	44.7
Ever tobacco use	Yes	66	10.3
	No	574	89.7
Ever Khat use	Yes	50	7.8
	No	590	92.2
Ever cannabis use	Yes	15	2.3
	No	625	97.7

### Psychosocial related factors

**Fig 1:**
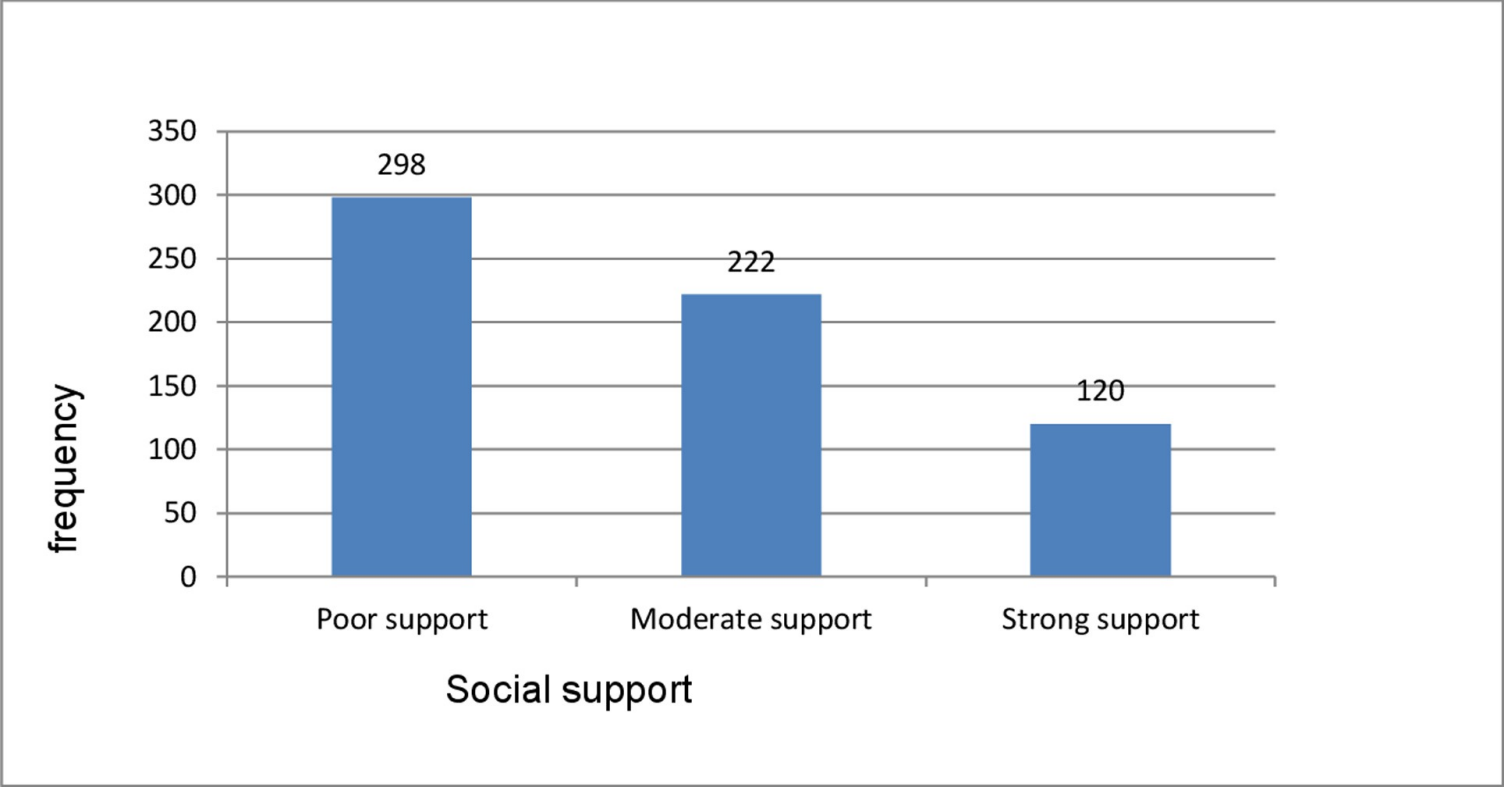
Frequency of social support among prisoners at Dilla correctional center, south, Ethiopia, 2020 (n = 640).

**Fig 2:**
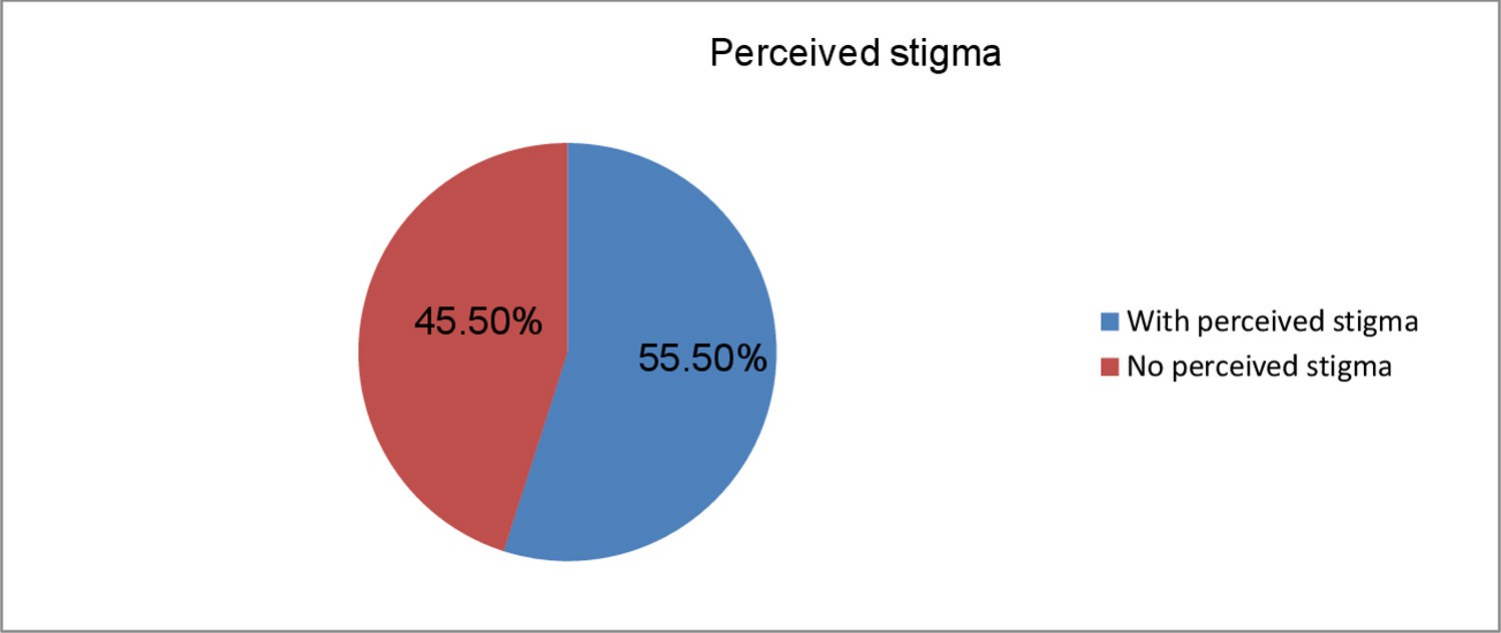
Frequency of perceived stigma among prisoners in Dilla town correctional center, south, Ethiopia, 2020 (n = 640).

Poor social support and perceived stigma were reported by 298 (46.6%) and 355 (55.5%) of the participants, respectively. (**Figs 1 and 2**)

### Magnitude of suicidal ideation and attempt

The lifetime prevalence of suicidal ideation of the participants was 140 (21.9%) with a 95% confidence interval of (95%CI: 18.4–25.2). Among those who had the history of suicidal ideation 68 (48.6%) had thought about committing suicide in the last one month and 60 (42.9%) had planned to commit suicide in their lifetime.

The lifetime prevalence of suicide attempts among participants was 84 (13.1%) with a 95% confidence interval of (95%CI, 10.6–15.8). Among these respondents, the proportion that had attempted suicide in the last month was 20 (23.8%). Of those who had a suicide attempts in their lifetime, 56 (66.7%) had made one attempt, 18 (21.4%) had made two attempts, and 10 (11.9%) had made more than two attempts.

The most commonly used method of the attempt was hanging 35 (41.7%) followed by poisoning 24 (28.6%). The major reasons for suicide attempt were hopelessness due to crime 35 (41.7%) and feeling guilty of crimes committed 24 (28.6%). Among the respondents who attempted suicide, 47 (56.0%) were seriously attempting to kill themselves, 29 (34.5%) tried to kill themselves, but the method they used was not foolproof. Only 8 (9.5%) individuals reported that the attempt was not a real intent to die but to seek help from the other **(Table 4).**

**Table 4:** Distribution of suicidal ideations and attempts among prisoners at Dilla town correctional center, south, Ethiopia, 2020 (n = 640).

Variable	Categories	Frequency	Percentage (%)
**Ever suicidal ideation**	**Yes**	140	21.9
	**No**	500	78.1
**Suicidal ideation in 1 month**	**Yes**	68	48.6
	**No**	72	51.4
**Ever plan of suicide**	**Yes**	60	42.9
	**No**	80	57.1
**Ever suicide attempt**	**Yes**	84	13.1
	**No**	556	86.9
**Suicide attempt in 1 month**	**Yes**	20	23.8
	**No**	64	76.2
**Frequency of suicide attempt**	**Once**	56	66.7
	**Twice**	18	21.4
	**More than two times**	10	11.9
**Reason for suicide attempt committed**	**Hopelessness due tocrime**	35	41.6
	**Feel guilty of crime**	24	28.6
	**Family conflict**	15	17.9
	**Economic problem/poverty**	10	11.9
**Severity related to attempt**			
	**Seriously attempted**	47	56
	**Methods used not effective**	29	34.5
	**To seek help**	8	9.5
**Methods of attempt**	**Hanging**	35	41.7
	**Poisoning**	24	28.6
	**Use sharp tools**	15	17.8
	**Other** *****	10	11.9

*****method = jump from high place and electricity.

### Factors associated with suicidal ideation

In multivariable logistic regression analysis, variables; female sex, divorce/widowed, family history of mental illness, common mental disorder, and poor social support were statistically significant with suicidal ideation at p-value less than 0.05.

The odds of having suicidal ideation among respondents who are females were 2.6 times higher compared to male respondents [AOR = 2.6, 95%CI (1.39, 8.2)].

The odds of having suicidal ideation among respondents who are divorced/ widowed were 3.67 times higher compared to married respondents [AOR = 3.67, 95%CI (2.05, 6.58)].

The odds of having suicidal ideation among respondents who had a family history of mental illness were 2.49 times higher as compared with those who had no family history of mental illness [AOR = 2.49, 95%CI, (1.41, 4.38)].

Those prisoners who had poor social support were more than two times more likely to have suicidal ideation compared to those who had strong social support [AOR = 2.68, 95%CI (1.42, 5.06)].

Participants who had a common mental disorders were 1.98 times more likely to have suicidal ideation than those who had not a common mental disorder [AOR = 1.98, 95%CI (1.25, 3.16)] **(Table 5).**

**Table 5:** Bivariate and multivariable logistic regression analysis, suicidal ideation, and associated factors among prisoners at Dilla town correctional center, south, Ethiopia, 2020 (n = 640).

Explanatory variables	Suicidal ideation		COR, (95%CI)	AOR, (95%CI)
	Yes	No		
**Sex**				
**Female**	25	37	2.53(1.49,6.3)	2.6(1.39, 8.2)**
**Male**	122	456	1	1
**Marital status**				
**Single**	56	270	1.00(0.64, 1.58)	1.17(0.72, 1.92)
**Divorced/widowed**	47	51	4.46(2.62,7.59)	3.67(2.05, 6.58)**
**Married**	37	179	1	1
**Family history of suicide attempt**				
**Yes**	10	28	1.41(0.85, 2.82)	1.23(0.67, 2.13)
**No**	122	482	1	1
**Family history of suicide commit**				
**Yes**	9	14	2.38(1.01, 5.63)	2.54(0.56, 6.69)
**No**	131	486	1	1
**Family history of mental illness**				
**Yes**	31	47	2.74(1.66,4.52)	2.49(1.41, 4.38)**
**No**	109	453	1	1
**Perceived stigma**				
**Yes**	87	268	1.42(0.97, 2.09)	1.34,(0.88, 2.05)
**No**	53	232	1	1
**Social support**				
**Poor**	83	215	2.34(1.32, 4.15)	2.68(1.42,5.06)**
**Moderate**	40	182	1.33(0.72, 2.47)	1.35(0.69, 2.65)
**Strong**	17	103	1	1
**Common mental disorder**				
**Yes**	102	288	1.98(1.31, 2.99)	1.98(1.25, 3.16)**
**No**	38	212	1	1
**Ever tobacco use**				
**Yes**	23	43	2.09(1.21, 3.61)	1.72(0.39,3.29)
**No**	117	457	1	1

### Factors associated with suicide attempt

From multivariable logistic regression variables; female sex, family history of mental illness, and previous incarceration were found to be statistically associated at p-value less than 0.05.

Being female was about 3.24 times more likely to have a suicide attempts compared to males [AOR = 3.24, 95%CI, (1.89, 9.4)].

The odds of having a suicide attempt among respondents who had a family history of mental illness were 2.08 times higher as compared with those who had not a family history of mental illness [AOR = 2.08,95%CI, (1.1, 3.9)].

Prisoners who had a previous incarceration were more than two times more likely to have a suicide attempts compared to those who had not previous incarceration [AOR = 2.38, 95%CI (1.2, 5.16)] **(Table 6).**

**Table 6:** Bivariate and multivariable logistic regression analysis suicide attempts among prisoners at Dilla town correctional center, south, Ethiopia, 2020 (n = 640).

Explanatory variables	Suicide attempt		COR, (95%CI)	AOR, (95%CI)
	Yes	No		
**Sex**				
**Female**	23	39	4.35(2.03,10.7)	3.24(1.89, 9.4)**
**Male**	69	509	1	1
**Marital status**				
**Single**	38	288	1.06(0.61, 1.82)	1.07(0.6, 1.89)
**divorced /widowed**	22	76	2.32(1.23,4.38)	1.9(0.87, 3.72)
**Married**	24	192	1	1
**Family history of suicide attempt**				
**Yes**	10	26	2.75(1.28, 5.94)	2.26(0.76, 5.76)
**No**	74	530	1	1
**Family history of suicide commit**				
**Yes**	5	18	1.95(1.12, 4.74)	1.28(0.78, 2.71)
**No**	77	540	1	1
**Family history of mental illness**				
**Yes**	19	59	2.46(1.38,4.39)	2.08(1.1, 3.9)*
**No**	65	497	1	1
**Ever Alcohol use**				
**Yes**	54	300	1.48(0.95, 2.47)	1.58(0.65, 2.62)
**No**	31	255	1	1
**Ever tobacco use**				
**Yes**	15	51	2.15(1.15, 4.04)	1.66(0.82, 3.34)
**No**	69	505	1	1
**Pervious incarceration**				
**Yes**	11	35	2.24(1.09, 4.61)	2.38(1.2, 5.16)*
**No**	73	521	1	1

1.00 = reference

*p-value less than 0.05

**p-value less than 0.01.

## Discussion

The study showed that the prevalence of suicidal ideation was 21.9% with (95%CI: - 18.4–25.2) and was similar with a study done in Pakistan 22% [25]. However, the finding of the current study was higher than studies done in Ethiopia, Jimma 16.6% [20] and in Addis Ababa 8.04% [26], On the other hand the finding of this study on the prevalence of suicide ideation among prisoners is lower than studies done in Belgium 43.1% [27], Australia 34% [14], Italy 43.7% [29], China 70% [28] and Iran 44.6% [29]. The possible reason for the variation might be the difference in the tool, which was the suicidal behavior questionnaire (SBQ-18 item) used in Jimma [20], 12-item suicidality scale from the Personality Assessment Inventory (PAI) was used in the USA and found that about 16% of the participants had clinically significant suicidal ideation [30] and Paykel suicidal scale (PSS), used in Belgium. A study done in North Russia found that the prevalence of suicidal ideation and suicidal attempt was 16.9% and 17.6% respectively [31]. The difference might be the study population in which only male inmates were involved. Another study done in Australia found that the lifetime prevalence of suicidal ideation was 33.7% and the suicidal attempt was 20.5% [14]. The possible reason for the variations may be the difference in sample size, sampling techniques ad sociocultural issues.

In the present study, females were 2.6 times more likely to have suicidal ideation than males. This is in agreement with the study conducted in England [32]. This could be due to psychosocial life stressors such as unpleasant life events, but it could also be due to socio-demographic or socio-economic factors, as well as sexual abuse that women face [33]. Furthermore, when confronted with adversity in their lives, women reported a stronger negative impact on their psychological well-being [34].

In this study, being divorced/ widowed were 3.67 times higher compared to married respondents. This is in line with the study conducted in Chicago [35]. Widowed status may be due to insinuating accomplice killings, which are related to the misfortune of control, envy, and relationship end frequently carried out within the warm of the minute [36]. In like manner, these detainees may be characterized by the next degree of impulsivity and maybe gone up against with strong sentiments of blame amid the consequent weeks of detention [37].

In the current study, those who had a family history of mental illness were 2.49 times more likely to have suicidal ideation compared with those who had not a family history of mental illness. This is supported by studies in Israel [38]. This might be individuals who had a family history of mental illness, may develop psychiatric disorder in family, and this may contribute to suicidal ideation.

In the present study, those who had a history of mental illness were 2.54 times more likely to have suicidal ideation than those who had not a history of mental illness. This is coinciding with results from Belgium, USA and New York [27, 30, 39]. This can be due to detainees who have a prior history of mental sickness are more likely to encounter trouble, blame feeling for their ailment, and need of social back, with numerous more stressors and substance use problems [40, 41].

In the current study, prisoners who had poor social support were 2.68 times more likely to have suicidal ideation than those who have strong social support. This is supported by other studies conducted in Belgium [27] and in North Russia [31]. This might be the fact that minimal friends inside or outside the correctional facilities, weak family support, and limited external contact are known to be major stressors for prisoners which contribute to suicidal ideation among incarcerated individuals.

Furthermore, in the study there is a strong relationship between common mental disorder and suicidal ideation. Participants who had a common mental disorders were 1.98 times more likely to have suicidal ideation than those who have no common mental disorder. This is in agreement with the study conducted in French [11]. The conceivable reason can be people who had common mental clutters are profoundly related to self-destructive ideation [40].

The current study showed that the prevalence of suicide attempts was 13.1% with (95%CI:- 10.6–15.8). This is comparable with a study done in Italy 12.8% [42]. On the other hand, the finding of the study was higher than study conducted at Jimma 9.3% [20]. However, the finding of this study was lower than studies conducted at Belgium 20.3% [27], Australia 21% [14] and Iran 38.9% [29]. The possible reason for the variation might be the sample size difference, in which 332 participants were involved and the tool used, in which the suicidal behavior questionnaire (SBQ-18) was used.

With respect to suicide attempts, being female was 3.24 times more likely to have suicidal attempts than males. This is in agreement with the studies conducted in Israel and India [36]. This could be due to females are more vulnerable to psychosocial life stressors such as unpleasant life events, but it could also be due to socio-demographic or socio-economic factors, as well as sexual abuse that they face [33]. Furthermore, when confronted with adversity in their lives, women reported a stronger negative impact on their psychological well-being [34].

In the present study, participants who had a family history of mental illness were 2.08 times higher as compared with those who did not have a family history of mental illness. This is supported by studies in Israel [36]. This may be individuals who have a family history of mental illness, may develop psychiatric disorders in family, and this may contribute to suicide attempts.

Prisoners who had a previous incarceration were 2.38 times higher compared to those who had not previous incarceration. This is supported by other similar studies conducted in Italy [43] and Jimma [20]. This might be due to prisoners with repeated imprisonment could face loneliness, loss of a spouse, and being deprived of important resources that could lead them to be at high risk [40].

### Limitations

Even though the study indicated essential factors associated with suicidal ideation and attempt, there are some limitations in this study. Since data collection was an interview method, it might face to social desirability bias because subjects were systematically more likely to provide a socially acceptable response. Since it is cross-sectional study design, it does not allow establishing a temporal relationship between suicidal ideation, attempt and significant associated factors. The international data are very limited to compare. For example, the age structure in prisons, the underlying laws, and also the suicide rates outside prisons differ considerably between countries.

## Conclusions

In this finding, the prevalence of suicidal ideation and attempt were high. Both suicidal ideation and attempt were associated with female sex and family history of mental illness, but being divorced/ widowed, poor social support, and common mental disorder was associated with suicidal ideation and previous incarceration was associated only with suicide attempts. The prison officer should integrate and strengthen prison mental health care services with Dilla University specialized referral Hospital and special attention for female prisoners, divorced/ widowed individuals, having poor social support, and prisoners with a history of previous incarceration.

## List of abbreviations

AOR: Adjusted Odds Ratio
CI: Confidence Interval
CIDI: Composite International Diagnostic Interview
COR: Crude Odds Ratio
WHO: World Health Organization
